# Chloramphenicol Induces Autophagy and Inhibits the Hypoxia Inducible Factor-1 Alpha Pathway in Non-Small Cell Lung Cancer Cells

**DOI:** 10.3390/ijms20010157

**Published:** 2019-01-03

**Authors:** Han-Lin Hsu, Po-Lin Liao, Yu-Wen Cheng, Shih-Hsuan Huang, Chien-Hua Wu, Ching-Hao Li, Jaw-Jou Kang

**Affiliations:** 1 Division of Pulmonary Medicine, Department of Internal Medicine, Taipei Medical University-Wan Fang Hospital, Taipei 116, Taiwan; lisa11011117@gmail.com; 2 Institute of Food Safety and Health Risk Assessment, School of Pharmaceutical Sciences, National Yang-Ming University, Taipei 110, Taiwan; plliao@ym.edu.tw; 3 School of Pharmacy, College of Pharmacy, Taipei Medical University, Taipei 110, Taiwan; ywcheng@tmu.edu.tw (Y.-W.C.); a001ou@gmail.com (S.-H.H.); 4 Institute of Toxicology, College of Medicine, National Taiwan University, Taipei 100, Taiwan; li.elfhaha@gmail.com; 5 Department of Physiology, School of Medicine; Graduate Institute of Medical Sciences, College of Medicine; Taipei Medical University, Taipei 110, Taiwan; 6 Institute of Food Safety and Health Risk Assessment, School of Pharmaceutical Sciences, National Yang-Ming University, Taipei 112, Taiwan

**Keywords:** chloramphenicol, Hypoxia inducible factor-1 alpha, Autophagy, SENP-1

## Abstract

Chloramphenicol is an inexpensive and excellent bactericidal antibiotic. It is used to combat anaerobic infections in the Third World countries, whereas its systemic application has been abandoned in developed countries. However, in recent years, clinicians have reintroduced chloramphenicol in clinical practice. In this study, chloramphenicol was found to repress the oxygen-labile transcription factor, hypoxia inducible factor-1 alpha (HIF-1α), in hypoxic A549 and H1299 cells. Furthermore, it suppressed the mRNA levels of vascular endothelial growth factor (VEGF) and glucose transporter 1, eventually decreasing VEGF release. Chloramphenicol initiated the autophagy pathway in treated cells, as observed by the increase in formation of Atg12-Atg5 conjugates, and in beclin-1 and LC3-II levels. The chloramphenicol-mediated HIF-1α degradation was completely reverted by autophagic flux blockage. In HIF-1α-overexpressing cells, the formation of HIF-1α/SENP-1 (Sentrin/SUMO-specific protease 1) protein complex seemed to facilitate the escape of HIF-1α from degradation. Chloramphenicol inhibited HIF-1α/SENP-1 protein interaction, thereby destabilizing HIF-1α protein. The enhancement in HIF-1α degradation due to chloramphenicol was evident during the incubation of the antibiotic before hypoxia and after HIF-1α accumulation. Since HIF-1α plays multiple roles in infections, inflammation, and cancer cell stemness, our findings suggest a potential clinical value of chloramphenicol in the treatment of these conditions.

## 1. Introduction

Hypoxic microenvironments exist frequently in the lungs of patients with airway diseases, especially chronic obstructive pulmonary diseases and tumors. Hypoxia-mediated cellular adaptations are dominantly orchestrated by the heterodimeric transcription factor hypoxia-inducible factor-1 (HIF-1). HIF-1 is comprised of one of three oxygen-sensitive α isoforms (HIF-1α, HIF-2α, and HIF-3α) and the constitutively expressed HIF-1β isoform (also called aryl hydrocarbon receptor nuclear translocator, ARNT) [1]. In a normoxia condition, HIF-1α is short-lived and is rapidly degraded. The activation of prolyl-4-hydroxylase (PHD) post-translationally modifies HIF-1α protein by hydroxylation, allowing the docking of the von Hippel–Lindau protein (pVHL), and subsequently directing HIF-1α degradation to the ubiquitin-proteasome system (UPS). On the contrary, the decline in PHD activity in response to oxygen deprivation results in the escape of HIF-1α from proteasomal degradation, leading to a rapid HIF-1α accumulation and activation [2,3]. For example, erythropoietin, glucose transporter-1 (GLUT-1) and vascular endothelial growth factor (VEGF) are well-known hypoxia-inducible genes. The hypoxia-responsive elements (HRE, with the core sequence of G/ACGTG) found in the promoter region of these genes, recruit the binding of HIF-1α, thereby activating these gene expression [4,5,6]. The upregulation of hypoxia-inducible genes is important for the processes that maintain the oxygen homeostasis, such as erythropoiesis, glucose metabolism, and angiogenesis [7,8].

Recently, it has been also accepted that there is a compensatory collaboration between the UPS and the autophagy-lysosome pathway, in HIF degradation [9]. Autophagy is a highly conserved, intracellular catabolic pathway, which occurs ubiquitously at basal level in all eukaryotic cells. The initiation of autophagic flux occurs in response to stress (e.g., starvation) and it basically fosters cell survival. Briefly, the long-lived proteins, protein aggregates, or aged/damaged organelles are engulfed by double-membrane vesicles, also called autophagosomes. The autophagosomes’ content is then digested to its basic components upon fusion with lysosomes, to sustain cellular metabolism and homeostasis [10]. The mechanism of autophagy was discovered in yeast and is mediated by genes referred to as autophagy-related (Atg) genes. Beclin 1 (mammalian ortholog of yeast Apg6) initiates the formation of isolation membrane [11]. The formation of Atg12-APG5 covalent conjugate (that is mediated by Atg7 and Atg10, and is ATP dependent) participates in the processing of LC3 (a homolog of yeast Atg8) to generate LC3-II, which contributes to the closure of the autophagosome [12]. Sequestosome 1 (also known as p62/SQSTM1) carries damaged proteins to the autophagosome [13]. Finally, the clusters of p62/SQSTM1 and ubiquitinated substrates, as well as LC3-II, are degraded by lysosomal hydrolases, when the autophagosome fuses with a lysosome.

Chloramphenicol was first isolated from the soil-borne bacteria, *Streptomyces venezuelae*, in 1947. It has excellent bioavailability and tissue bio-distribution, regardless of the administration route [14,15]. Crystallography studies on bacteria showed that chloramphenicol efficiently bound to the peptidyl transferase center of the bacterial 50S ribosomal subunit, blocking the aminoacyl-tRNA moiety and inhibiting protein translation [16]. Thus, it is effective against a broad spectrum of pathogenic bacteria, and thereby, cures infections. It was popular until the late 1980s. Then, chloramphenicol oral therapy was hampered due to epidemiological reports of rare but serious adverse effects associated with hematologic suppression [17]. On the other hand, the low cost and the ease of chemical synthesis of chloramphenicol are currently linking again this drug with an increasing number of human medications in many developing countries, to combat typhoid fever, anaerobic infections, meningitis, brain abscesses, and rickettsial infections. Antibiotic resistance has been rapidly burgeoning worldwide in the past few decades, and it outpaces the development of new antimicrobial compounds. The revisit of old or abandoned antimicrobial drugs, especially those the use of which was reduced because of their toxic side effects, is considered as an important strategy. Among these disputed antibiotics, chloramphenicol attracts special attention [18,19,20], especially with respect to multiple drug resistant (MDR) bacteria [18]. In this study, we initially found that chloramphenicol induced autophagy and inhibited the protein interaction between HIF-1α and Sentrin/SUMO-specific protease 1 (SENP-1). Both these actions promoted HIF-1α protein degradation and offered a potential therapeutic effect against hypoxia-associated diseases.

## 2. Results

### 2.1. Short Incubation with Chloramphenicol Does Not Affect Cell Viability in Normoxia or Hypoxia Conditions

A549 and H1299 cells were incubated with chloramphenicol (1-100 μg/mL) for 3 h and 24 h, and assayed for their viability. In the 3-h-treated group, the viability of A549 and H1299 cells at 100 μg/mL concentration was 97.0 ± 3.9% and 98.1 ± 5.0%, respectively. Next, after pre-incubation with chloramphenicol for 3 h, cells were subjected to hypoxic conditions for 8 h or treated with 250 μM CoCl_2_. The viability of A549 cells was 102.9 ± 1.3% and 99.2 ± 0.9%, whereas the viability of H1299 cells was 103.3 ± 1.9% and 93.8 ± 4.5%, under hypoxia and treatment with CoCl_2_, respectively (Table 1). Therefore, no cytotoxicity was observed in these testing conditions. However, a significant reduction in cell viability was observed in the 24-h-treated groups. Overall, the cell viability of the testing conditions throughout the study were over 80%. 

### 2.2. Chloramphenicol Inhibited HIF-1α Protein Accumulation in Non-Small Cell Lung Cancer (NSCLC) Cells in a Concentration-Dependent Manner

**Figure 1:**
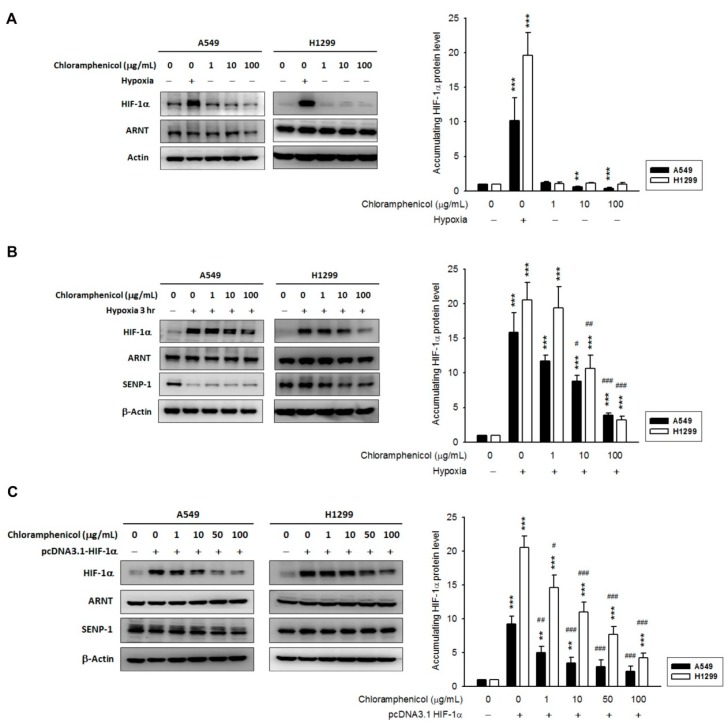
Chloramphenicol inhibited HIF-1α protein accumulation in NSCLC cells in a concentration-dependent manner. (**A**) In a normoxia condition, the expression of HIF-1α protein in H1299 cells was not unaffected by chloramphenicol (1–100 μg/mL) treatment, whereas in A549, the basal HIF-1α protein level was reduced. A 3 h hypoxic treatment was used as positive control. *N* = 3. (**B**) In both cell lines, the hypoxia-induced HIF-1α protein accumulation was significantly inhibited by chloramphenicol (a 3-h pretreatment, followed by incubation under hypoxic conditions for another 3-h). A repression of SENP-1 levels was observed in hypoxia-treated cells. *N* = 3 (A549) and 5 (H1299). (**C**) In A549 and H1299 cells, with an overexpression of HIF-1α (by transient transfection of pcDNA3.1 HIF-1α), exposed to chloramphenicol for 18-24 h, the level of HIF-1α protein was significantly reduced compared to vehicle control. ARNT and SENP-1 levels remained unaltered. *N* = 3 (A549) and 5 (H1299). Quantification data was generated via densitometric analysis from at least three independent experiments and presented as mean ± S.E. (* *p* < 0.05, ** *p* < 0.01, and *** *p* < 0.001 indicates statistically significant difference from the control group; ^#^ *p* < 0.05, ^##^ *p* < 0.01 and ^###^ *p* < 0.001 indicates statistically significant difference from the hypoxia-treated control).

Hypoxia-exposed A549 and H1299 cells showed high levels of HIF-1α protein accumulation within 3 h. The treatment with chloramphenicol alone (18–24 h) did not change the HIF-1α protein level in non-hypoxic H1299 cells, whereas it reduced the HIF-1α protein basal level in A549 (Figure 1A). Interestingly, in both hypoxic cell lines, the pre-incubation with chloramphenicol (1–100 μg/mL) for 3 h significantly inhibited HIF-1α protein accumulation induced by hypoxia (Figure 1B), while the expression levels of ARNT remained unaltered. In hypoxic cells, we also found reduced levels of SENP-1 compared to non-hypoxic cells (Supplementary Figure S1). It was observed that CoCl_2_ could stabilize HIF-1α levels and could mimic the effects of hypoxia mimicry by generating a transcriptional profile in cells similar to that induced by hypoxia [21]. CoCl_2_ is widely used as chemical hypoxia mimetic. Unexpectedly, chloramphenicol had no effect on CoCl_2_ (250 μM, 3 h treatment)-mediated HIF-1α protein accumulation and SENP-1 protein reduction. Moreover, the expression of ARNT was potentiated by hypoxia in A549 cells, but not in H1299 cells (Supplementary Figure S2).

To elucidate the direct effect of chloramphenicol on HIF-1α protein stability, HIF-1α-overexpressing A549 and H1299 cells were established. The amount of HIF-1α protein was significantly reduced in chloramphenicol-treated groups compared to the vehicle-treated control group, whereas there was no significant change in the levels of ARNT and SENP-1 (Figure 1C). Moreover, chloramphenicol treatment had no effects on the protein levels of H1299 cells overexpressing p53 or aryl hydrocarbon receptor (AHR) proteins (Supplementary Figure S3). These data suggest that chloramphenicol might specifically inhibit HIF-1α protein accumulation, either by incubation before hypoxia or after HIF-1α accumulation. 

### 2.3. Hypoxia-Induced mRNA Expression of GLUT-1 and VEGF, and the Secretion of VEGF Protein Were Inhibited by Chloramphenicol Treatment

**Figure 2:**
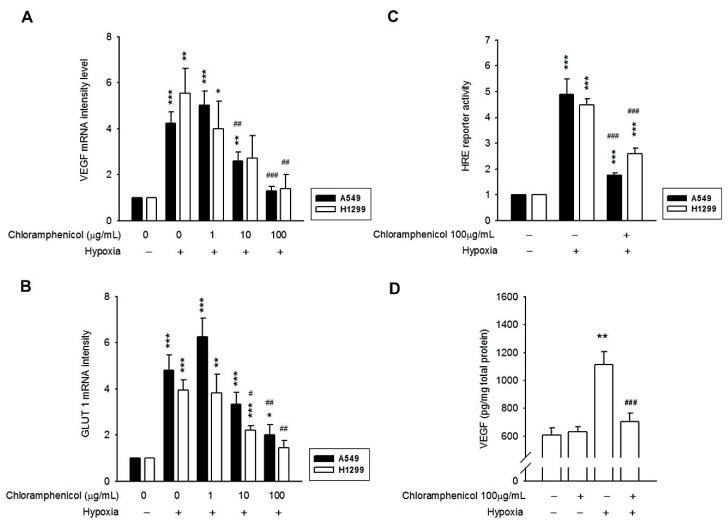
Chloramphenicol inhibited the HIF-1α pathway in NSCLC cells. The induction of VEGF mRNA (**A**), GLUT-1 mRNA (**B**), HRE reporter (**C**), and VEGF (**D**) protein secretion were confirmed after the hypoxia treatment; whereas, these inductions were reversed by chloramphenicol treatment. (* *p* < 0.05, ** *p* < 0.01, and *** *p* < 0.001 indicates statistically significant difference from the control group; ^#^ *p* < 0.05, ^##^ *p* < 0.01, and ^###^ *p* < 0.001 indicates statistically significant difference from the hypoxia-treated control). (**A**,**B**) *N* = 4 (A549) and 5 (H1299); (**C**) *N* = 6 (A549) and 11 (H1299); (**D**) *N* = 6.

GLUT-1 and VEGF are well-known hypoxia-inducible genes that could be activated directly via the binding of HIF-1α to HREs in their promoter [4,5,6]. However, in the chloramphenicol-pretreated group, under a hypoxia condition, VEGF and GLUT-1 mRNA expressions were significantly inhibited (Figure 2A,B). Hypoxia-induced HRE reporter transactivation was also suppressed by chloramphenicol (Figure 2C, Supplementary Figure S4). The conditioned medium collected from the previous treatments was quantified for secreted VEGF, and the results showed that VEGF secretion was upregulated in hypoxia treated cells, but was significantly suppressed after chloramphenicol treatment (Figure 2D). These data prove that chloramphenicol not only reduces HIF-1α protein accumulation but also represses the downstream effects of HIF-1α. 

### 2.4. Chloramphenicol Promoted HIF-1α Protein Degradation Via Autophagy-Dependent Pathway

**Figure 3:**
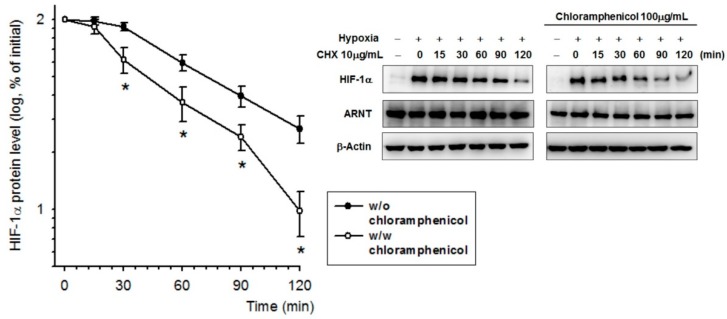
Chloramphenicol destabilized the HIF-1α protein. HIF-1α is a short-lived protein. A representative cycloheximide-chase assay for evaluating HIF-1α turnover in the presence of chloramphenicol is shown at the top, and the quantitative results of multiple experiments (probit model) are shown below. After a 3-h treatment under a hypoxia condition for HIF-1α protein induction, cells were shifted to a normoxia incubator prior to the cycloheximide-chase assay. The amount of HIF-1α present at the given chase time was expressed relative to that observed at time zero. We found that the half-life (t_1/2_) of HIF-1α protein was reduced from 78.2 min (without chloramphenicol preincubation) to 55 min (with chloramphenicol preincubation). (* *p* < 0.05 indicates a statistically significant difference from the hypoxia-treated control). *N* = 5.

HIF-1α protein stability was evaluated in the presence of chloramphenicol using a cycloheximide chase assay. The half-life (t_1/2_) of HIF-1α protein after hypoxia exposure was reduced from 78.2 min (without chloramphenicol preincubation) to 55 min (with chloramphenicol preincubation) (Figure 3). Thus, the data reveal that chloramphenicol might destabilize HIF-1α protein promoting its earlier degradation.

**Figure 4:**
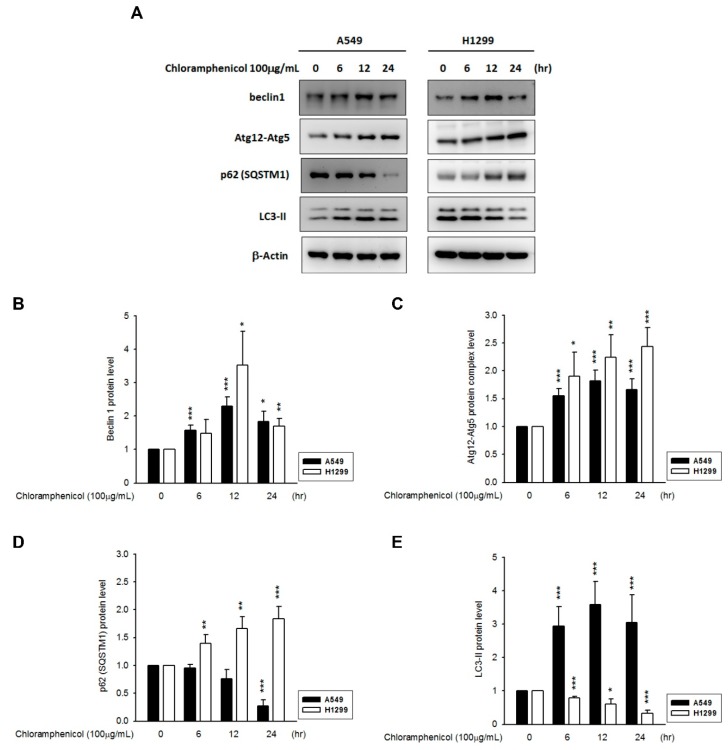
Chloramphenicol induced autophagy in NSCLC cells in a time-dependent manner. (**A**) Representative images showing the changes in levels of autophagy biomarkers (beclin 1, Atg12-Atg5 conjugates, p62/SQSTM1, and LC3-II) in A549 and H1299 cells in response to chloramphenicol treatment. Quantitative results of multiple experiments are shown for (**B**) beclin 1, (**C**) Atg12-Atg5 conjugates, (**D**) p62/STSQM1, and (**E**) LC3-II. (* *p* < 0.05, ** *p* < 0.01, and *** *p* < 0.001 indicates statistically significant difference from the control group). *N* = 5 (A549) and 3 (H1299).

**Figure 5:**
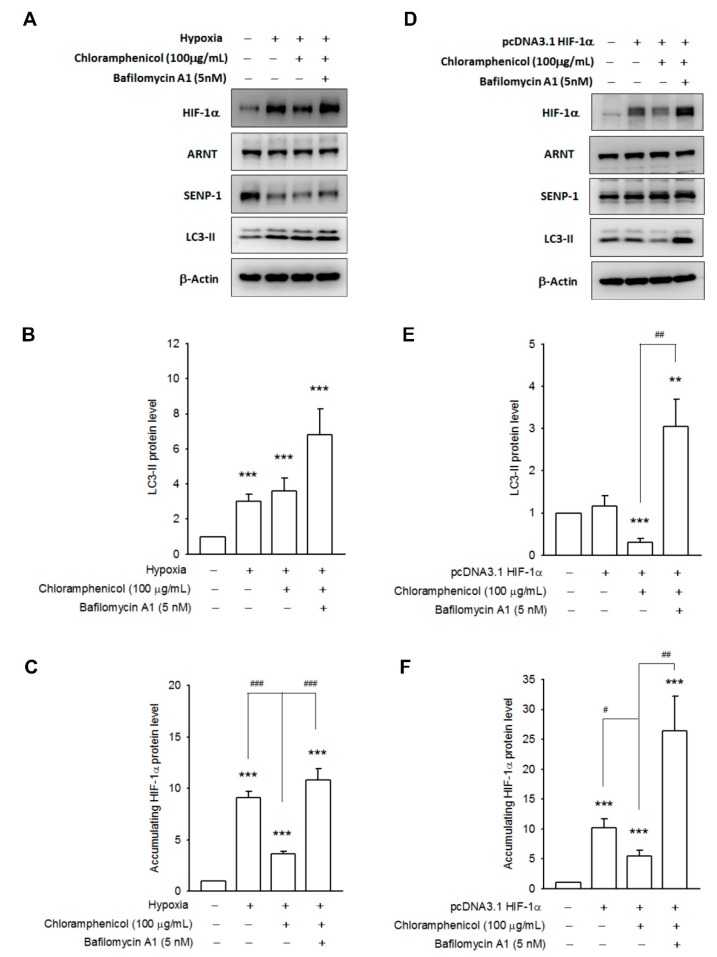
Chloramphenicol potentiated HIF-1α protein degradation via the autophagy pathway. Representative images showing the inhibition of autophagic flux by bafilomycin A1 (5 nM), and the amelioration of chloramphenicol-mediated HIF-1α degradation in hypoxia-conditioned A549 cells (**A**) or in HIF-1α-overexpressing H1299 cells (**D**). Quantitative results of multiple experiments are shown for (**B**,**E**) LC3-II, and (**C**,**F**) HIF-1α. (*** *p* < 0.001 indicates statistically significant difference from the control group; ^#^ *p* < 0.01, ^##^ *p* < 0.01 and ^###^ *p* < 0.001 indicates statistically significant difference from the chloramphenicol-treated control). *N* = 4 (A549 and H1299).

Recent reports have indicated that hypoxia can induce autophagy [22], and UPS and autophagy are associated with HIF-1α protein degradation. Thus, we investigated the expression profiles of autophagy proteins (beclin-1, Atg12-Atg5 conjugates, p62/SQSTM1, and LC3-II) in both chloramphenicol-treated NSCLC cell lines. We found that chloramphenicol upregulated the expression of beclin-1 and increased the levels of Atg12-Atg5 conjugates in both NSCLC cell lines, both in a time dependent (Figure 4A–C) and concentration-dependent manner (Supplementary Figure S5A–C). In A549 cells, LC3-II was augmented after chloramphenicol treatment, whereas p62/STSQM1 was downregulated. Both of these events indicate initiation of the autophagosome formation and autophagic digestion. Interestingly, in H1299 cells, which are characterized by a higher basal autophagic flux [23], chloramphenicol induced an augmentation of p62/STSQM1, which in turn, functioned as cargo receptor and facilitated the delivery of ubiquitinated proteins to the autophagosome, and a decrease in LC3-II levels (Figure 4D,E; Supplementary Figure S5D,E). The decrease in LC3-II levels in H1299 represented a faster autophagic flux and digestion. This could be demonstrated through a pretreatment with bafilomycin A1 (5 nM), which blocks the fusion between autophagosomes and lysosomes, and prevents the maturation of autophagic vacuoles. Bafilomycin A1 not only fully restored the LC3-II levels in chloramphenicol-treated H1299 cells, but also potentiated the LC3-II accumulation in A549 (Figure 5A,B) and H1299 cells (Figure 5D,E). The chloramphenicol-mediated prevention of HIF-1α protein accumulation induced by hypoxia, was completely ameliorated in bafilomycin A1-treated A549 cells (Figure 5C), whereas the expression patterns of ARNT and SENP-1 remained unaltered (Supplementary Figure S6). Co-incubation with bafilomycin A1 inhibited the chloramphenicol-mediated HIF-1α degradation in HIF-1α overexpressing H1299 cells (Figure 5F), whereas the expression levels of ARNT and SENP-1 remained unaltered (Supplementary Figure S6). These data strongly demonstrate that chloramphenicol-mediated HIF-1α protein degradation is mainly driven by the autophagy pathway. 

### 2.5. Chloramphenicol Interfered with Protein Interaction between SENP-1 and HIF-1α to Promote HIF-1α Degradation via Autophagy Pathway

**Figure 6:**
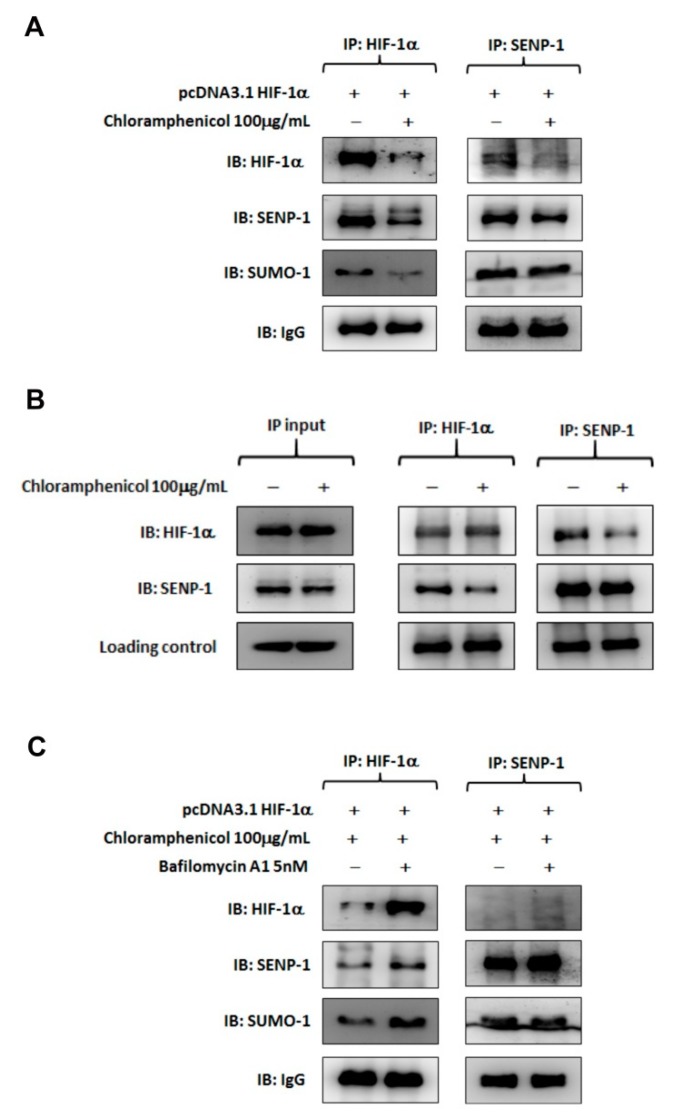
Chloramphenicol interrupted protein interaction between SENP-1 and HIF-1α and promoted HIF-1α degradation via the autophagy pathway. (**A**) Representative images of co-immunoprecipitation assays performed with HIF-1α-overexpressing H1299 cells in the presence or absence of chloramphenicol. Whole cell lysates was immunoprecipitated with anti-HIF-1α (left) or anti-SENP-1 (right) antibody, followed by immunoblotting analysis. A protein complex of HIF-1α/SENP-1 was found in the control treatment. Chloramphenicol treatment not only reduced HIF-1α levels in whole lysate, but also inhibited HIF-1α/SENP-1 interaction. (**B**) Whole lysate of HIF-1α-overexpressing H1299 was prepared and aliquoted (1 mg protein/vial). Before reaction with the captured antibody, one vial of sample was incubated with chloramphenicol (100 μg/mL) at 4 °C for 1 h. Another vial, without chloramphenicol treatment, was used as a control. Then, the co-immunoprecipitation was performed as described previously. The “input” data showed that neither HIF-1**α** nor SENP-1 protein level was changed in whole lysates, whatever the presence or absence of chloramphenicol treatment. The HIF-1α/SENP-1 protein complex, which was clearly found in lysate without the chloramphenicol treatment, was diminished in lysate with the chloramphenicol treatment. This data provides substantial evidence to support the interaction between HIF-1α and SENP-1 was damaged by chloramphenicol. (**C**) Bafilomycin A1 inhibited the HIF-1α protein degradation induced by chloramphenicol; however, there was still no generation of the HIF-1α/SENP-1 protein complexes. *N* = 4.

By using co-immunoprecipitation with a capture antibody specific to either HIF-1α or SENP-1, the HIF-1α/SENP-1 protein complexes were detected in HIF-1α-overexpressing H1299 cells. SENP-1 was able to regulate HIF-1α levels via deSUMOylation, thus, resulting in stabilization and transactivation of HIF-1α [24]. Chloramphenicol treatment disrupted this interaction and directed HIF-1α toward degradation as the result of reduction of HIF-1α/SENP-1 complexes (Figure 6A,B). Co-incubation with bafilomycin A1 restored HIF-1α protein levels, but the HIF-1α/SENP-1 interaction remained impaired. Especially for protein complexes precipitated by the capture antibody specific to SENP-1, the co-precipitated HIF-1α was diminished in bafilomycin A1 treated group compared to the protein complexes precipitated by the capture antibody specific to HIF-1α (Figure 6C). These data indicated that chloramphenicol-mediated HIF-1α protein degradation was induced by the disruption of HIF-1α/SENP-1 interaction and promotion of autophagy dependent degradation.

## 3. Discussion

The significant role of HIF-1α in determining the outcome of cancer progression has been well documented [1,25,26]. Hypoxia not only facilitates VEGF and platelet-derived growth factor (PDGF) expression in the HIF-1α-dependent pathway, but also contributes to the development of chemo-resistance [27,28,29]. In addition, emerging evidence has implicated HIF-1α in infectious and inflammatory diseases among different tissues. The stabilization of HIF and the induction of hypoxia responsive genes occur in a wide range of infections, including bacteria (e.g., *Staphylococcus aureus*, *Bartonella henselae*, *Escherichia coli*, *Chlamydia trachomatis*, *Pseudomonas aeruginosa*, etc.), viruses (e.g., hepatitis C, varicella–zoster virus, human herpes virus, etc.), and *Candida albicans* [7,8], and distinct mechanisms might be involved. For example, multiplication of microbial cells deprives them of oxygen and ATP. Some microbial factors (such as lipopolysaccharide and siderophores) have been implicated in HIF-1α stabilization. In the presence of infections, hypoxia not only suppresses the innate immune response [30], but also reinforces the production of proinflammatory cytokines (e.g., TNF-α, IL-1β, IL-6, etc.), and matrix metalloproteinase-1 [7,31,32,33]. The positive feedback loop between hypoxia and inflammation often exacerbates the inflamed lesions and may initiate early sepsis. The progression of lipopolysaccharide-induced sepsis is less severe in HIF-1α-deficient mice, as compared to that in wild-type [33]. Thus, the inhibition of HIF-1α expression or activity may offer a therapeutic potential against infections, inflammation and cancer progression. 

In this study, we found that chloramphenicol is a potent inhibitor of HIF-1α, either by incubation before hypoxia or after HIF-1α accumulation. However, chloramphenicol did not prevent the accumulation of HIF-1α induced by CoCl_2_. The biochemical mechanism of HIF-1α stabilization mediated by CoCl_2_ is controversial. It has been validated that CoCl_2_ could stabilize HIF-1α protein levels either by inactivation of PHD through the replacement of iron from its iron-binding center, or by direct inhibition of the interaction between von Hippel–Lindau protein (pVHL) and hydroxylated HIF-1α [34]. A pVHL-independent ubiquitination of HIF-1α at the N-terminal region induced by CoCl_2_ has been reported to resist proteasomal degradation [35]. Several signaling molecules, such as the phosphatidylinositol-3 kinase (PI-3K) and mitogen activated protein (MAP) kinases, which are implicated in the accumulation of polyubiquitinated proteins, could also be quantified by CoCl_2_ [36]. It is more likely that CoCl_2_ as well as other cobalt complexes are potent inhibitors of UPS [37,38,39]. Multi-pathways induced by CoCl_2_ converge towards the accumulation of HIF-1α protein, especially towards the direct inhibition on the proteasome activity, and thus may be the reason of the inactivity of chloramphenicol on HIF-1α degradation, upon CoCl_2_ exposure.

In this study, we also found that the promotion of HIF-1α degradation by chloramphenicol essentially involved autophagy. Chloramphenicol treatment substantially increased the levels of autophagic biomarkers (beclin-1, Atg12-Atg5 conjugates, and LC3-II), which suggested an initiation of autophagosome formation. The movement of autophagic flux can be validated using bafilomycin A1, which inhibits the autophagosome fusion with a lysosome and reinforces LC3-II accumulation. Recently, several studies supported the autophagy-mediated degradation of HIF-1α. Q6, a novel hypoxia-targeted agent, promotes HIF-1α degradation and attenuates the kinetics of HIF-1α downstream genes. Both of these functions of Q6 could be reversed either by using short interfering RNA (siRNA) targeting Atg5 and LC3 or by using bafilomycin A1. In addition, HIF-1α was further found to interact with p62/SQSTM1, indicating autophagic degradation [40]. Autophagy induced by the β-2 adrenergic receptor antagonists ICI118551 and butoxamine [41] or by the inhibitor of mammalian targets of rapamycin complex 1/2 (mTORC1/2) AZD-2014 [42], which also leads to the autophagic degradation of HIF-1α, reduction in glucose uptake and glycolysis, and reduced cell vitality. Chloroquine, a lysosomotropic agent which acts as an inhibitor of autophagy at the late stage, potentiated HIF-1α accumulation [43]. In our study, bafilomycin A1 treatment not only restored chloramphenicol-mediated HIF-1α degradation but also enhanced HIF-1α protein accumulation, suggesting that HIF-1α is, in part, constitutively degraded by autophagy.

Aside from the PHD-mediated hydroxylation, other post-translational modifications of HIF-1α, such as acetylation, phosphorylation, and SUMOylation contribute to HIF-1α stability [44]. SUMOylation is dynamically catalyzed by an enzymatic cascade that facilitates the activation (E1), conjugation (E2), and ligation (E3) of small ubiquitin-like modifier (SUMO) to the lysine residues of the target proteins. In general, the SUMOylated protein, which changes its function and subcellular localization, is implicated in a variety of cellular physiology reactions. A family of Sentrin/SUMO-specific proteases (SENPs) removes the SUMO from SUMOylated protein by a process called deSUMOylation. In mammalian cells, six SENPs with differences in sequence homology, subcellular distribution, and substrate specificity have been identified [45,46,47]. SENP-1, with a broad substrate specificity, deSUMOylates HIF-1α. In the absence of SENP-1, HIF-1α is actively SUMOylated and degraded, even under hypoxic conditions [24]. Consistently, hypoxia-induced VEGF, GLUT-1, and heme oxygenase-1 are suppressed by SENP-1 knockdown [48,49]. In this study, by using co-immunoprecipitation, we first found that overexpressed HIF-1α coupled to SENP-1 prevented HIF-1α decay. Chloramphenicol treatment blocked the formation of the HIF-1α/SENP-1 protein complex, thereby promoting HIF-1α degradation. 

Overall, it was disclosed for the first time that chloramphenicol initiates autophagic flux, compromises HIF-1α/SENP-1 complex formation, enhances HIF-1α protein degradation, and finally inhibits hypoxia responsive gene transactivation. The function of chloramphenicol as a HIF-1α inhibitor provides new insights into hypoxia-related treatments.

## 4. Materials and Methods 

### 4.1. Cell Culture

The NSCLC cell line, A549 was purchased from Bioresource Collection and Research Center (BCRC, Hsinchu, Taiwan), and the cell line, H1299 was a gift from Dr. Pan Shiow-Lin (College of Medical Science and Technology, Taipei Medical University, Taipei, Taiwan). Both cell lines were cultured in DMEM supplemented with 10% fetal bovine serum (FBS), 2 mM L-glutamine, 200 U/mL penicillin, and 200 μg/mL streptomycin (Life Technologies, Carlsbad, CA, USA) at 37 °C in a 5% CO_2_ atmosphere. 

### 4.2. Hypoxia Condition

For inducing hypoxia, cells were placed in a hypoxic chamber (Anaerobic system ProOx model 110, BioSpherix, Lacona, NY, USA), set at providing less than 1% O_2_ for indicated time. Control groups were placed under normoxic incubator for equivalent periods. To mimic hypoxic condition, cells were plated on a 60-mm dish and were treated with CoCl_2_ (250 μM, Fluka Biochemika Ltd., Mexico City, Mexico) for indicated periods under a 5% CO_2_ atmosphere at 37 °C.

### 4.3. Cell Viability Assay

Cell viability was evaluated using MTT [3-(4,5-dimethyl-2-thiazolyl)-2,5-diphenyl-2H- tetrazolium bromide] assay according to a previous study [50]. Briefly, 2 × 10^4^ cells/well were plated in 48-well culture dish at a final volume of 0.5 mL/well, and after 24 h, cells were exposed to different conditions as indicated. After the addition of MTT reagent (final concentration of 100 μg/mL), cells were further incubated for 2 h. Then, the metabolites of MTT were eluted thoroughly using DMSO. We then measured the absorbance of the samples at 570 nm against the control group, using a multiwall plate reader (Chromate 4300, Awareness Technology, Inc., Palm City, FL, USA).

### 4.4. Western Blot Analysis

After treatment, cells were washed with cold PBS, harvested, and lysed in radioimmunoprecipitation assay (RIPA) buffer containing protease and phosphatase inhibitors as described previously [50]. The lysates were centrifuged at 14,000 rpm for 20 min at 4 °C, and the protein concentration of the supernatant was determined using a Bradford protein assay kit (Bio-Rad Laboratories Inc., Hercules, CA, USA) according to the manufacturer’s instructions. Protein samples were mixed with 4× Laemmli sample buffer (62.5 mM Tris-HCl (pH 6.8), 10% glycerol, 1% SDS, and 0.005% bromophenol blue), and equal amounts (30 μg/well) of protein were then subjected to 10 or 15% sodium dodecyl sulfate-polyacrylamide gel electrophoresis (SDS-PAGE) at 90–100 V for 2 h. Next, proteins in a separating gel were transferred to polyvinylidene difluoride (PVDF) membranes at 400 mA for 40 min. After being blocked with 5% non-fat milk in Tris Buffered Saline with Tween 20 [TBST, 150 mM NaCl, 10 mM Tris (pH 7.5), and 0.05% Tween-20] at room temperature for 30 min, the blots then incubated overnight at 4 °C with the following primary antibodies: anti-HIF-1α, anti-Atg12, anti-LC3 (GeneTex, Inc., Irvine, CA), anti-SENP-1 (Abcam, Inc., Cambridge, MA, USA), anti-ARNT, anti-p62/SQSTM1, anti-beclin 1, anti-SUMO1 (Santa Cruz, Inc., Heidelberg, Germany), and anti-β-Actin (Sigma-Aldrich, St. Louis, MO, USA). Following a 30 min washing with TBST, the blots were then hybridized for 2 h at room temperature with the secondary antibody conjugated with horseradish peroxidase (HRP). Finally, protein signals were visualized with an enhanced chemiluminescence (ECL) kit (Millipore, Billerica, MA, USA) and images were captured using the BioSpectrum AC^®^ Imaging system (UVP, Upland, CA, USA). Protein signals were quantified using the Gel-Pro analyzer software (version 4.0, Media Cybernetics, Rockville, MD, USA). Background intensity was subtracted from each sample and the sample signal was normalized to the β-actin loading control. 

### 4.5. Reverse Transcription and Quantitative Polymerase Chain Reaction (RT-qPCR)

Total RNA was isolated using TRIzol isolation kit (Thermo Fisher Scientific, Inc.) as previously described [50]. Equal amounts of RNA were reverse transcribed with MMLV Reverse Transcriptase (BioGenesis, Taiwan) to cDNA. Quantitative reverse transcription polymerase chain reaction (qRT-PCR) was performed on the resulting cDNA using OmicsGreen qPCR Master Mix with ROX dye (Omicsbio, Taiwan) according to the manufacturer’s instructions. The thermal cycling conditions used for the PCR were as follows: 95 °C for 10 min, followed by 45 cycles of 95 °C for 30 s, 60 °C for 30 s, and 72 °C for 30 s. Relative levels of gene expression were quantified using the 2^−ΔΔ*C*q^ method using LightCycler^®^Nano (Roche Molecular Systems, Inc., Almere, Flevoland, Nederland). Glyceraldehyde-3-phosphate dehydrogenase (GAPDH) was used as a normalization of for mRNA levels and each sample was analyzed in triplicates. Primer sets of GAPDH, VEGF, and GLUT1 are listed in Supplementary Table S1.

### 4.6. Enzyme Linked Immunosorbent Assay (ELISA)

Conditioned medium was harvested from NSCLC cell cultures subjected to different treatment. VEGF levels secreted in the medium were quantified using ELISA kits according to the manufacturer’s protocol (R&D systems, Minneapolis, MN, USA). Absorbance was measured at 405 nm on a microplate reader (BioTek Instruments, Inc., Winooski, VT, USA). VEGF concentrations (pg/mg total protein) were calculated from a standard curve. 

### 4.7. Transfection

The pcDNA3.1 plasmid expressing wild-type HIF-1α was obtained from Addgene (Cambridge, MA, USA). Transfection into H1299 cells was performed using TurbofectTM transfection reagent (Thermo Scientific, Waltham, MA, USA). 

### 4.8. Luciferase Reporter Assay

The HRE luciferase reporter construct (pGL2-HRE) and pRK5-LacZ were co-transfected as described previously [50]. Twenty-four hours after transfection, cells were exposed to hypoxia (or CoCl_2_). Then, the cell extracts were harvested and luciferase activity was assayed using the reporter assay system (Promega, Madison, WI, USA).

### 4.9. Co-Immunoprecipitation

Whole cell lysates were pre-cleared via a short incubation with protein A magnetic beads (Millipore). Then, the captured antibodies (1 μg/mL) were added into the pre-cleared lysates (1 mg) and incubated overnight at 4 °C with rotation. The captured antibodies used in this study were antibodies against HIF-1α (Thermo Fisher, catalog No. PA116601) and SENP-1 (Abcam, catalog No. ab108981). The next day, 5 μL protein A magnetic beads was added and incubated with shaking for another 2 h. The captured immunocomplexes were precipitated and washed three times with RIPA buffer prior to the addition of 100 µL of 2× SDS sample buffer and heating at 95 °C for 5 min. Samples were separated on 10% SDS-PAGE and analyzed as described earlier. 

### 4.10. Statistical Analysis

Results are presented as the mean ± S.E. and comparisons between multiple groups were evaluated using one-way ANOVA followed by Duncan’s new multiple comparison using SPSS software (version 18.0, SPSS Inc., Chicago, IL, USA). The significance of the differences between the control and each experimental test condition were analyzed using Student’s *t*-tests. *p* < 0.05 was considered statistically significant. Each experiment included at least three replicates per condition. 

## Figures and Tables

**Table 1:** Effects of chloramphenicol on non-small cell lung cancer cell viability.

Cell Type		Chloramphenicol (g/mL), 3 h Incubation				Chloramphenicol (g/mL), 24 h Incubation			
		0	1	10	100	0	1	10	100
**Cell viability (%)**	**A549**	100.0 ± 1.0	103.6 ± 2.3	95.6 ± 1.5	97.0 ± 3.9	100.0 ± 0.8	96.0 ± 1.1 *	92.4 ± 1.4 ***	89.3 ± 1.4 ***
	**H1299**	100.0 ± 0.9	101.1 ± 1.3	98.3 ± 1.5	98.1 ± 5.0	100.0 ± 2.4	97.5 ± 1.8 **	93.8 ± 2.1 ***	92.4 ± 2.5 ***
**Chloramphenicol treatment followed by an 8 h hypoxia condition**									
**Cell viability (%)**	**A549**	100.0 ± 0.8	102.0 ± 3.6	99.1 ± 1.6	102.9 ± 1.3	100.0 ± 0.4	92.4 ± 1.0 ***	85.6 ± 1.0 ***	81.0 ± 0.8 ***
	**H1299**	100.0 ± 1.0	101.3 ± 0.9	98.7 ± 0.9	103.3 ± 1.9	100.0 ± 1.7	98.5 ± 0.8	98.1 ± 1.6	93.4 ± 1.6 ***
**Chloramphenicol treatment followed by an 8 h CoCl_2_ (250 M) incubation**									
**Cell viability (%)**	**A549**	100.0 ± 1.5	101.1 ± 1.9	97.4 ± 1.2	99.2 ± 0.9	100.0 ± 1.1	98.4 ± 1.7	93.0 ± 2.2 *	77.0 ± 1.1 ***
	**H1299**	100.0 ± 0.9	101.8 ± 1.1	95.3 ± 1.6	93.8 ± 4.5	100.0 ± 1.2	100.6 ± 2.5	99.8 ± 2.0	88.5 ± 4.1 ***

^1^ Cell viability was evaluated by using the MTT assay as described in Materials and Methods. Data shown here were generated from at least three independent assays (*N* = 4–6). ^2^ * *p* < 0.05, ** *p* < 0.01, and *** *p* < 0.001 indicates a statistically significant difference from the control group.

## Supplementary Materials

Supplementary materials can be found at http://www.mdpi.com/1422-0067/20/1/157/s1.

## Abbreviations

HIF-1α	Hypoxia inducible factor-1 alpha
NSCLC	Non-small cell lung cancer
VEGF	Vascular endothelial growth factor
PHD	Prolyl hydroxylase
pVHL	von Hippel–Lindau protein
ARNT	Aryl hydrocarbon receptor nuclear translocator
HREs	Hypoxia response elements
RT-PCR	Reverse transcription-polymerase chain reaction
GLUT-1	Glucose transporter 1
PDGF	Platelet-derived growth factor
UPS	Ubiquitin-proteasome system
SUMO	Small ubiquitin-like modifier
SENP-1	Sentrin/SUMO-specific protease 1
