# Supplementary material for: Chloramphenicol Induces Autophagy and Inhibits the Hypoxia Inducible Factor-1 Alpha Pathway in Non-Small Cell Lung Cancer Cells
Source: Int J Mol Sci. 2019 Jan 3;20(1):157. doi: 10.3390/ijms20010157 (PMC6337541; doi:10.3390/ijms20010157)
Supplement: Supplementary file 1 [file ijms-20-00157-s001.pdf]

Supplementary Information

Supplementary Table S1. Primer sets for qPCR.

Gene	Sense primer	Antisense primer
VEGF	GAGATGAGCTTCCTACAGCAC	TCACCGCCTCGGCTTGTCACAT
GLUT-1	CCCGCTTCCTGCTCATCAA	GACCTTCTTCTCCCGCATCATC
GAPDH	GAAGGTGAAGGTCGGAGTCAAC	CAGAGTTAAAAGCAGCCCTGGT

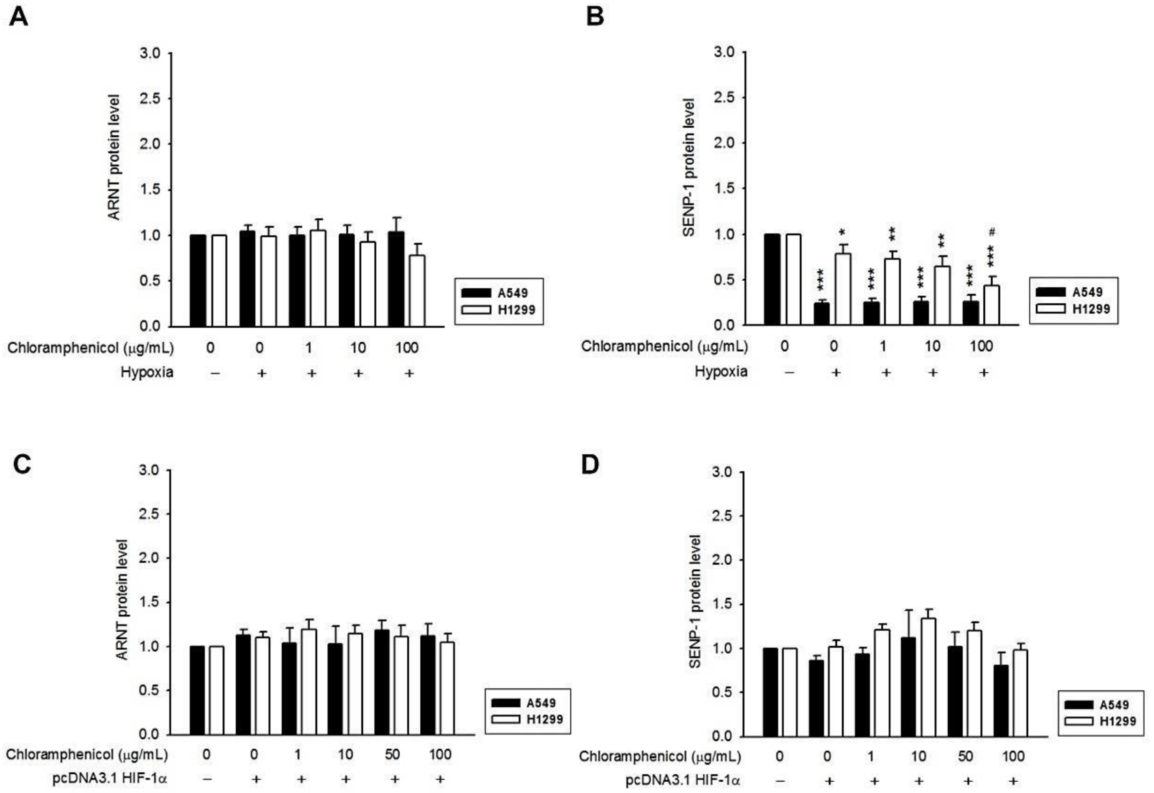

Supplementary Figure S1. Quantification data of ARNT and SENP-1 proteins in NSCLC cells after chloramphenicol and hypoxia treatments. Representative images of Western blotting were showed in Figure 1B and 1C. Quantification data of ARNT (**A** and **C**) and SENP-1 (**B** and **D**) indicated that chloramphenicol had no obvious effects on their expression. However, hypoxia treatment (3 h) did reduce SENP-1 content (* $p < 0.05$, ** $p < 0.01$, and *** $p < 0.001$ indicates a statistically significant difference from the control group; # $p < 0.05$ indicates a statistically significant difference from the hypoxia-treated control).

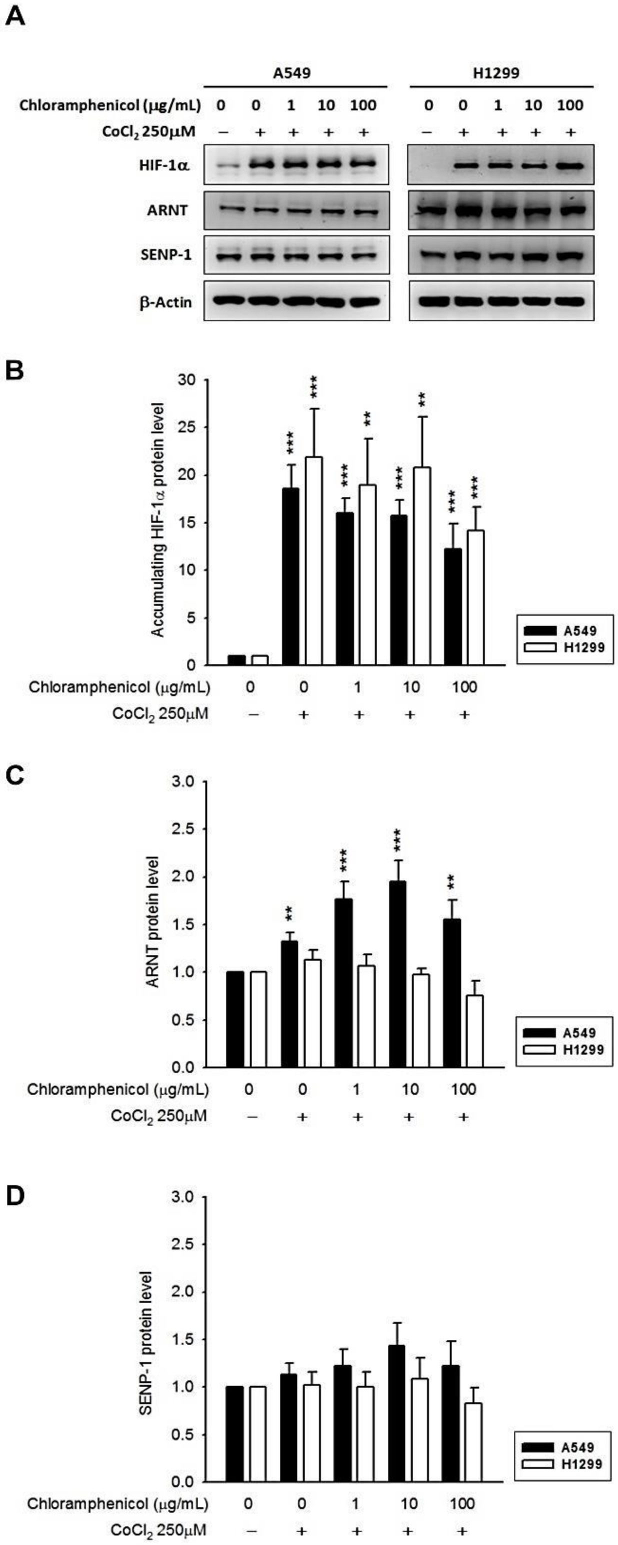

Supplementary Figure S2. Chloramphenicol had no effect on $CoCl_2$-mediated HIF-1◎ accumulation. (**A**) Images showed that $CoCl_2$ (250 M, 3 h) treatment caused an increase in HIF-1◎ protein level, which could not be prevented by chloramphenicol pre-incubation. In A549, the expression of ARNT was also upregulated by hypoxia and potentiated by chloramphenicol co-incubation. SENP-1 was without significant changes. Quantified data of HIF-1◎ (**B**), ARNT (**C**) and SENP-1 (**D**) was generated by densitometry analysis. (* $p < 0.05$, ** $p < 0.01$, and *** $p < 0.001$ indicates a statistically significant difference from the control group). $N = 5$ (A549) and 3 (H1299).

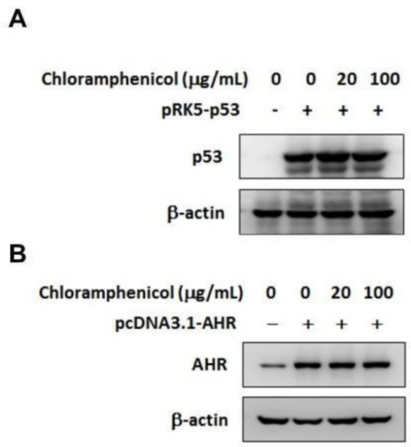

Supplementary Figure S3. Chloramphenicol did not down-regulate the ectopic expression of p53 and the acyl hydrocarbon receptor (AHR) in H1299. H1299 was p53-null and AHR poor-expressed, thus an overexpression of p53 and AHR could be observed easily. The amount of p53 (**A**) and AHR (**B**) proteins were without differences between chloramphenicol treated groups and vehicle control, suggested that HIF-1 was a unique target of chloramphenicol.

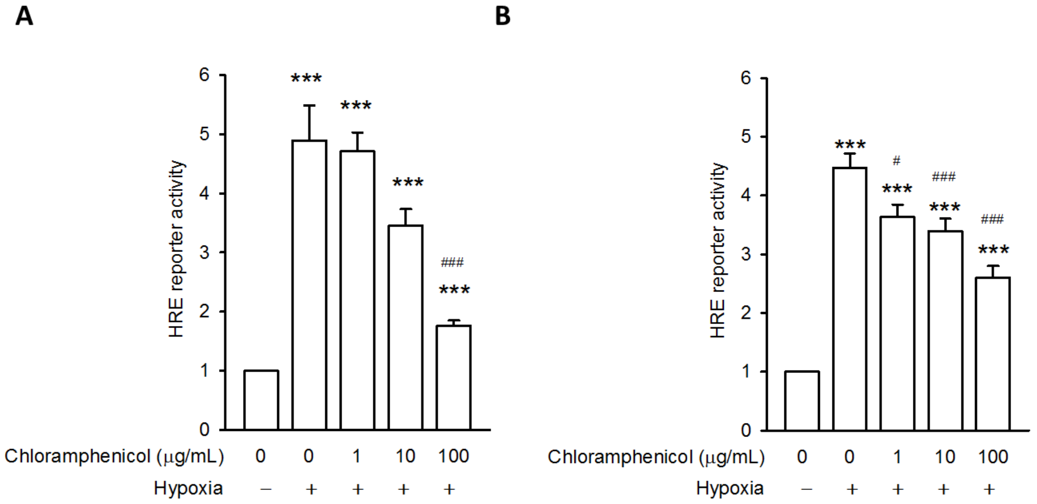

Supplementary Figure S4. Chloramphenicol diminished the HRE reporter activity induced by hypoxia. Cells were co-transfected with 2 μg of the HRE luciferase reporter construct (pGL2-HRE) together with 0.5 μg of pRK5-LacZ. After 24 h of transfection, cells were exposed to a hypoxia condition for 6–9 h, and then, cell extracts were harvested. Luciferase activity was measured by using the reporter assay system (Promega, Madison, WI, USA). (*** $p < 0.001$ indicates a statistically significant difference from the control group; # $p < 0.01$, and ### $p < 0.001$ indicates statistically significant difference from the hypoxia-treated control). In Figure 2C, only the highest treatment was shown. (**A**) A549, N = 6; (**B**) H1299, N = 11.

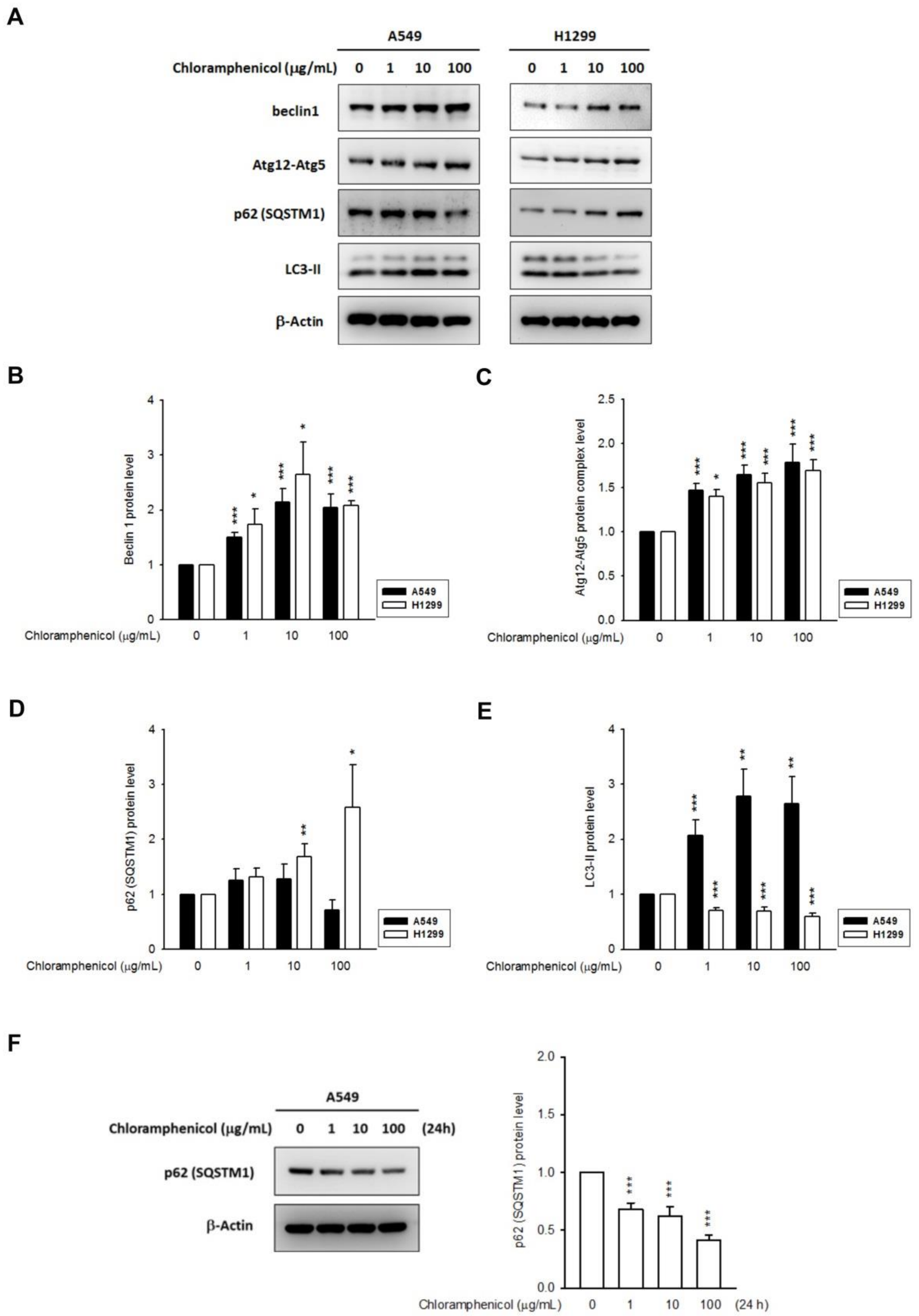

Supplementary Figure S5. Chloramphenicol initiated autophagy in NSCLC cells in a concentration-dependent manner. (**A**) Representative images showed the changes of autophagy biomarkers (beclin 1, Atg12-Atg5 conjugates, p62/SQSTM1, and LC3-II) of A549 and H1299 in response to a 12-h chloramphenicol treatment. Quantitative results of multiple experiments are shown as (**B**) beclin 1, (**C**) Atg12-Atg5 conjugates, (**D**) p62/STSQM1, and (**E**) LC3-II. (* $p < 0.05$, ** $p < 0.01$, and *** $p < 0.001$ indicates a statistically significant difference from the control group). (**F**) A clear concentration-dependent degradation of p62/SQSTM1 was observed in A549 after 24 h of chloramphenicol treatment. N = 4 (A549 and H1299).

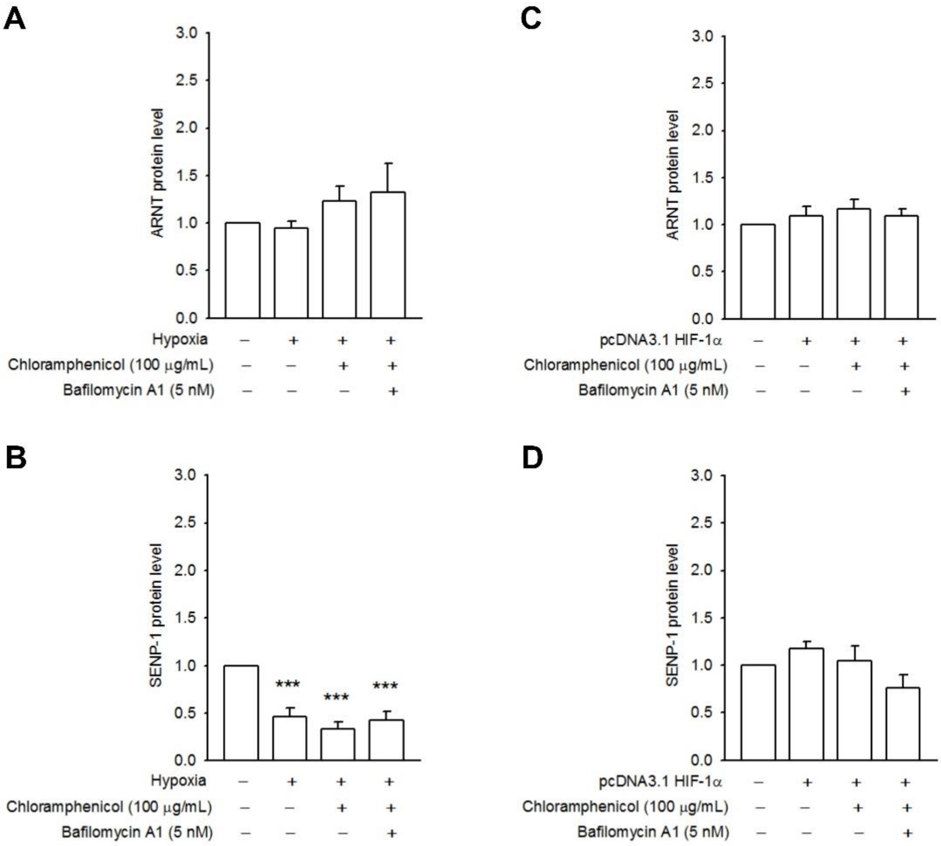

Supplementary Figure S6. Quantitative results of Figure 5. Quantitative results of multiple experiments are shown for ARNT (**A**/**C**) and SENP-1 (**B**/**D**). (*** p < 0.001 indicates statistically significant difference from the control group). N = 4 (A549 and H1299).
